# Biomechanical evaluation of midwifery tasks and its relationship with the prevalence of musculoskeletal disorders

**DOI:** 10.1016/j.heliyon.2023.e19442

**Published:** 2023-08-28

**Authors:** Naser Hasheminejad, Maryam Amirmahani, Somayeh Tahernejad

**Affiliations:** Department of Occupational Health Engineering and Safety at Work, School of Public Health, Kerman University of Medical Sciences, Kerman, Iran

**Keywords:** 3DSSPP, Work-related musculoskeletal disorders

## Abstract

**Introduction:**

As an important occupational group in the healthcare system, midwives face various ergonomic risk factors such as musculoskeletal disorders (MSDs) risks. Moreover, an accurate job evaluation can help to design appropriate ergonomic interventions and more accurately estimate the probability of developing MSDs. Therefore, the present study aimed to investigate musculoskeletal discomforts and biomechanical stresses using the Three-Dimensional Static Strength Prediction Program (3DSSPP Ver. 7.1.3) to find the association of these factors with the prevalence of MSDs among midwives.

**Materials and methods:**

A total of 91 midwives with at least two years of work experience participated in this cross-sectional descriptive study. All midwifery duties were analyzed using the hierarchical task analysis (HTA) method. Moreover, time analysis was performed for this job, and data were collected using the Nordic Musculoskeletal Questionnaire (NMQ) and body mapping. The 3DSSPP software was used to investigate the forces acting on the lumbar region, and finally, the association of individual characteristics and the forces exerted on the body with MSDs was investigated.

**Results:**

According to the results, the highest level of pain and discomfort was related to the back and neck regions. The software analysis of the four major midwifery tasks showed that the highest biomechanical forces were exerted on the L_5_/S_1_ disc during lifting the mothers from bed (to help them sit or walk) and breastfeeding training. Accordingly, only in the task of lifting the mothers and helping them to sit or walk, the balance status was inappropriate and critical due to the improper distribution of forces. The analysis of the associations between variables showed that biomechanical forces, age, height, body mass index, and job tenure were among the risk factors affecting MSDs.

**Conclusion:**

According to the results of this study, MSDs were highly prevalent, especially in the neck and back regions; this can be attributed to the nature of midwifery tasks. The software analysis results of the tasks showed that the biomechanical forces on the spine in each of the tasks can be affected by the weight of the mother, the height of the bed, static posture, and the bending/twisting of the whole body. Compression and shear forces were among the risk factors that can affect MSDs. To prevent MSDs in midwives, it is appropriate to improve the workstations.

## 1 Introduction

Musculoskeletal disorders (MSDs) are one of the most common occupational diseases and a major cause of the reduction in ability and productivity of the workforce. As an important occupational group in the healthcare system, midwives are exposed to many physical and psychological stressors [1]. Midwives manage heavier-than-average patients and help move/lift the patients after epidural anesthesia. Due to the nature of midwifery, midwives often maintain prolonged awkward postures and perform tasks that can increase the risk of MSDs. The importance of this subject and the high frequency of MSDs among midwives necessitate immediate action to reduce MSDs in this occupational group [2].

The underlying cause of work-related MSDs is not currently known. However, one assumption is that MSDs are caused by cumulative micro damages due to cellular- or tissue-level risk factors over time [3]. Some studies have shown that compared to prolonged exposure to ergonomic risk factors, the application of excessive force to different areas of the body is more effective in developing MSDs [4].

The risk of developing back disorders can be evaluated by the maximum compression force exerted on the lumbar intervertebral discs [5]. It is believed that excessive compression and shear forces acting on the spine or joints can lead to MSDs [6].

Many studies have investigated the prevalence of MSDs in various groups of healthcare workers [[7], [8], [9]]. The annual prevalence of work-related low back pain (LBP) among healthcare workers is estimated to be 55% [10]. However, there are relatively few studies in this field conducted on midwives [11].

According to a study conducted in Australia, the prevalence of work-related MSDs in midwives was 40.8% in the neck region and 24.5% in the back region. These results are comparable to work-related MSD rates in nurses and doctors [12,13]. Studies have shown that there is a strong association between the MSD risk and certain positions for lifting mothers from bed (e.g., twisting and bending of the spine). These results indicate that frequently helping patients is associated with a higher risk of pain and musculoskeletal injuries among healthcare workers [14,15]. Among the studies conducted on occupational health in midwives, only a few have focused on the ergonomics of midwifery tasks. Okuyucu et al. evaluated the midwifery tasks using the Rapid Entire Body Assessment (REBA) method and estimated the risk of MSDs to be very high among midwives. However, REBA and other observational ergonomics assessment methods that have been used for midwives rely only on screening and do not provide an accurate estimation of the biomechanical stresses on the body of the subjects [16].

To the best of our knowledge, no study has been found that uses biomechanical software or objective tools to investigate the biomechanical stresses on the body of midwives during their tasks. Estimating the forces on different joints of the body are valuable findings that have not been presented in any study so far. Considering that biomechanical forces are one of the effective factors in the occurrence of musculoskeletal disorders. These findings are valuable because if we want to perform an ergonomic intervention in midwives, we can use the data of biomechanical forces on the joints and perform the desired intervention in such a way that the amount of forces in the joints is reduced.

In addition, the relationship between biomechanical forces and the prevalence of musculoskeletal disorders, which has not been investigated in the target group of midwives, can be another strength and value of this study. Our data can be new findings that will be very helpful in designing appropriate ergonomic interventions, more accurate estimation of the probability of MSDs and more accurate job evaluation.

Therefore, the present study was conducted with the aim of investigating the following issues.

• Determining the prevalence of musculoskeletal disorders among midwives
• Calculation of forces on different parts of the body using the Three-Dimensional Static Strength Prediction Program (3D SSPP Ver. 7.1.3)
• Determining the relationship between the force applied to each area of the body and musculoskeletal disorders in the corresponding area

The findings of this study can probably be used as an important source of information for occupational health managers in the field of midwifery and for providing more effective interventions and ergonomic training to reduce the prevalence of MSDs in midwives.

## 2 Materials and methods

### 2.1 Participants

This cross-sectional descriptive study was conducted in August and September 2020. In this study, 91 participants were recruited from midwives working in the maternity hospitals of Kerman, Iran, with at least two years of work experience. The participants were selected for the study through the census method. Accordingly, every midwife who met the inclusion criteria entered the study voluntarily. The exclusion criteria were taking on a second job that may lead to MSDs and having MSDs due to an accident or any other activity except midwifery. All participants signed a written informed consent before the study. This study was approved by the Ethics Committee of Kerman University of Medical Sciences via ethics code No.IR.KMU.REC.1399.154. In addition, all participants gave their informed consent for the publication of their photographs in scientific journals through a written form.

### 2.2 Data gathering tools

#### 2.2.1 Demographic questionnaire

This questionnaire included age, weight, height, work experience, work shifts, second job, marital status, and educational level.

#### 2.2.2 Nordic musculoskeletal questionnaire

The General Nordic Musculoskeletal Questionnaire (NMQ) was developed in 1987 by Kournica et al. to be used in the screening of musculoskeletal disorders as part of ergonomic programs in epidemiological studies. This Questionnaire examines the reported cases of MSDs in different body parts, including nine anatomical regions (neck, shoulders, upper back, lower back, hands, feet, knees, thighs, and elbows) separately for left and right sides. This information either prompts further in-depth research or provides tips for deciding on preventative measures [17]. In this study, the Persian version of the NMQ questionnaire was used. Its psychometric properties were evaluated by Choobineh et al. and the correlation coefficient of more than 0.65 indicates the acceptable reliability of the questionnaire [18].

#### 2.2.3 Hierarchical task analysis

Hierarchical task analysis (HTA) was proposed by Annett and Duncan in 1967 for educational purposes [19]. The HTA method is a well-known task analysis method and an original ergonomic approach, which has been used for more than 30 years [20]. In this method, activities are described in terms of goals, sub goals, operations, and plans. The final result provides a comprehensive description of the activities [21].

#### 2.2.4 3D SSPP program

The 3DSSPP software Ver. 7.1.3 was used to investigate the forces acting on the lumbar spine. This software has been developed for 40 years in the Center for Ergonomics at the University of Michigan to examine static and biomechanical forces based on the physical needs of the work environments [22]. Indeed, 3DSSPP is one of the most widely used ergonomic computer programs, which can simulate the posture of individuals in various activities [23].

The 3DSSPP can aid the ergonomics analyst as a design and evaluation tool in both proactive and reactive analysis of workplaces and work tasks. 3DSSPP can assist the ergonomic analyst as a design and evaluation tool in the active and reactive analysis of workplaces and work tasks [22]. The results of this software show that it is a very suitable software for ergonomic and biomechanical evaluation (analysing the movement of organs and applying force in different tissues of the body) [24].

### 2.3 Procedure

In the first stage of the present study, after investigating the occupational features of midwifery, interviewing midwives, and observing the midwifery tasks separately, the HTA method was used to analyze all the tasks. Additionally, time analysis was performed for this job.

**Fig. 1:**
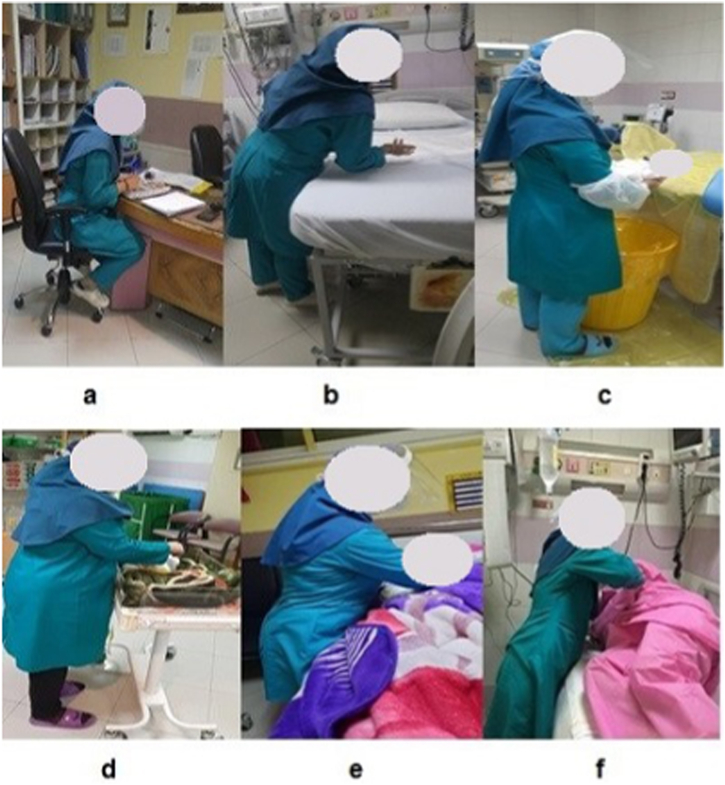
Some sample photographs of midwifery tasks and posture of midwives in maternity wards (a: filling the maternity notes folders; b: performing vaginal examination; c: delivery bed status and posture of handling the newborn; d: placing the newborn in warmer for drying; e: breastfeeding training; f: lifting the mothers from bed to sit or walk).

**Fig. 2:**
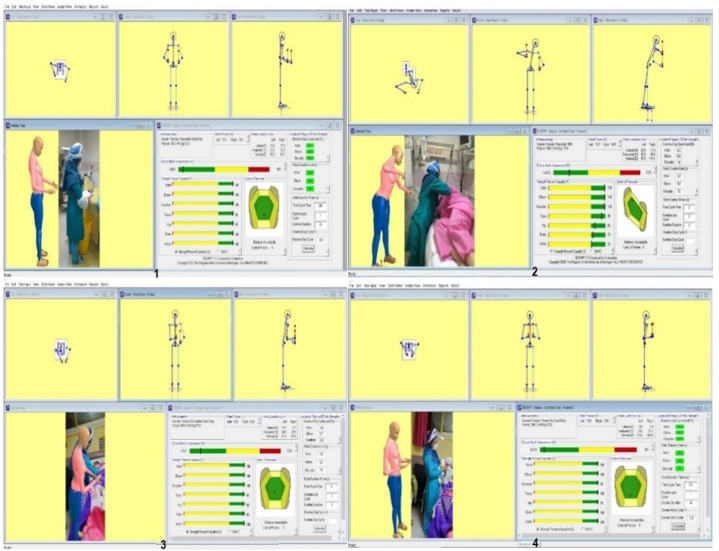
Sample images of the simulated and analyzed postures in the software environment (1: Analysis of delivery bed status and posture of handling the newborn; 2: Analysis of lifting the mothers from bed to sit or walk; 3: Analysis of breastfeeding training; 4: Analysis of holding and handling newborns).

The prevalence of MSDs was assessed using NMQ, and body mapping was used to identify different body regions. The researcher visited the participants' workplaces and explained how to complete the questionnaire, and the questionnaires were then completed by the participants. In this study, HTA is used to analyze complex tasks by dividing work into subtasks and describing steps and objectives. To select the postures that should be analyzed using 3D SSPP software, a video camera was used and observation was done in different work cycles. As a result, the most important tasks and inappropriate postures were selected. If the work cycle was long, the evaluation was done at specific time intervals. Of course, the length of time a person stays in a posture was also considered. It should be noted that photos or videos of work postures were taken from 3 angles: side, front and back. Some examples of photographs of midwives' posture while performing midwifery duties in the maternity hospital are presented in Fig. 1. Then, software simulation yielded the compression and shear forces at the L_5_/S_1_ disc. Moreover, forces on other body parts were calculated, and the body balance was evaluated. In Fig. 2, Sample images of the simulated and analyzed postures in 3D SSPP software environment can be seen.

### 2.4 Statistical analysis

To analyze the collected data, SPSS Statistics 20 was used. Descriptive statistical methods were used to show the characteristics of the study subjects. An independent *t*-test was used to determine the relationship between the forces acting on the lumbar spine and MSDs, while both independent *t*-test and chi-square test were used to determine the relationship between individual factors and MSDs. The significance level was set at p < 0.05.

## 3 Results

### 3.1 Findings related to demographic characteristics and HTA

A total of 91 midwives participated in this study. Eighty-two percent of the participants had a bachelor's degree and about 30% of them had less than five years of work experience. The average age of the participants was 35.70 ± 6.089 years, and 84% of them were shift workers. The average weight of newborns was 2.991 ± 0.117 and the average weight of mothers was 70.109 ± 2.979. Other demographic details are presented in Table 1.

**Table 1:** Demographic/Population data.

Characteristics	n(%)/Mean ± SD
Age (years)	35/7 ± 6.089
Age <29	21 (23.1%)
Age 30-40	57 (62.6%)
Age >41	13 (14.3%)
Height(cm)	161.03 ± 5.111
Weight(kg)	68.4 ± 11.706
BMI	26/395 ± 6/089
Underweight = BMI <18.5	2 (2.2%)
Normal weight = 18.5–24.9	40 (44%)
Overweight = 25–29.9	26 (28.6%)
Obesity = BMI ≥30	23 (25.3%)
Job tenure (years)	9/93 ± 5/416
<5	27 (29.7%)
5–10	28 (30.8%)
≥10	36 (39.5%)
Marital status	
Single	25 (27.5%)
Married	66 (72.5%)
Educational level	
Associate Degree	1 (1.1%)
Associate	82 (90.1%)
BSc and above	8 (8.8%)
Work schedule	
Day-work	15 (16%)
Shift-work	76 (84%)

The most important midwifery duties, which required a significant amount of time, were determined according to the results of HTA. These duties included the birth process from the start and handling the baby until the removal of the placenta (35%), perineal suturing and preparing the mother to be transferred to the postpartum room (30%), and breastfeeding training and lifting the mother to sit or walk in the postpartum room (20%). The average time intervals required for these activities were as follows: birth process 45 min, perineal suturing 30 min, proper breastfeeding training 20 min, and lifting the mother to sit or walk 15 min. The other information can be seen in Table 2.

**Table 2:** Hierarchical analysis of midwife duties in maternity hospital.

Duty	task	Subtask	Time	Workload
Admission of pregnant woman	description	Checking height, weight, records of previous births and blood transfusions, mental-psychological-social health and special diseases if any	40 min	4%
	Giving clothes to pregnant women to enter labor unit			
Training on arrival	Familiarity with the department and introduction of personnel and treatment methods			
Examination and control of vital signs of pregnant woman	Control of pulse, blood pressure, breathing and temperature	Controlling pulse and blood pressure using pulse oximeter and monitor		
		Counting Respiration		
		Checking armpit temperature with thermometer		
Fetal ECG monitoring	Using the NST device through the surface of the mother's abdomen			
Attaching peripheral venous catheter and sampling for testing	Attaching peripheral venous catheter	Blood sampling		
Entering the pain room				
Vaginal manual examinations according to the stage of labor	Pain control by midwives		20 min	10%
	Control of rupture of the water bag (ROM)and bleeding		2 min	
	Tachycardia and bradycardia control with Sonicad device	Controlling the progress of labor and how the fetus is placed to determine the position of labor	2 min	
Training of breathing techniques and exercises during labor to progress childbirth			30 min	2%
Massage therapy			30 min	2%
Arriving at the beginning of labor and transferring the mother to the delivery room	Use a wheelchair if needed		1 min	3%
Bed adjustment	Adjusting the height of the bed in the area between the elbow and the shoulder for the midwife to dominate the fetus using control			
hand Washing and wearing boots, face shield, Sterile gown and gloves			2 min	
Washing and disinfecting the birthing area			1 min	
Spreading Surgical Drapes (around the birthing area)			1 min	
Lidocaine injection if needed			1 min	Almost 35% of the work
Episiotomy if needed	Cutting the perineum			
Beginning of labor	Being in the right position to control the fetal head and prevent perineal rupture		5 min	Almost 30% of the work
	Taking the baby		30 min	
	skin-to-skin contact with the mother			
	umbilical cord clamping			
	umbilical cord blood collection			
	Cleaning the baby's nasal and throat secretions			
	Putting the baby in the warmer and drying it	Drying and removing wet towels	10 min	
		Assessment of the baby's condition and keeping the head warm with a hat		
		Diapering and dressing the baby		
	Measuring height, weight and head circumference of the baby			
Placenta leaving the uterus	Helping to expel the placenta after placental abruption		5–10 min	
Adjust the light manually			2 min	
Suturing of the rupture of the perineum			20 min	
Vital signs control			5 min	
Uterine massage			3 min	
Transfer to postpartum room	Wheelchair transfer		1 min	Almost 20% of work
	Oxytocin injection	Injection in the serum	1 min	
	Breastfeeding help		20 min	
	Vitamin K injection to the baby		1 min	
	Control vital signs and bleeding up to 2 h		Every 15 min total 30 min	
	Help the patient sit and set up	Helping to sit on the edge of the bed	15 min	
		Helping the mother to walk		
Moved to the women's department				

### 3.2 Prevalence of MSDs

The results of the NMQ showed that 96.7% of the participants had reported at least one case of MSD during the past 12 months with the highest frequency in the neck and lower back area. Accordingly, 33% of the midwives had been on sick leave due to the pain and discomfort in the lower back region and 26.4% due to the pain and discomfort in the neck region, during the past 12 months.

Some of the participants reported more than one MSD case during the past 12 months. Among the participants, 29.7% of people reported MSD in two body parts at the same time while 27.5% reported four or more MSDs. Moreover, only 3.3% of midwives had no disorders.

### 3.3 Posture analysis using 3DSSPP

The common postures for the four selected duties were evaluated based on HTA, time analysis, and the output of 3D SSPP. The results showed that for the task of lifting the mothers from bed (to help them sit or walk), the highest compression and shear forces with averages of respectively 417.246 ± 022.2346 and 322.29 ± 35.155 were exerted on the lower back region (at L_5_/S_1_).

Moreover, the lowest force was exerted on the joints of the hands and wrists during perineal suturing, and the highest force was exerted on the left ankle joint when helping to lift the mother from the bed. Table 3 shows the forces exerted on the other body parts while performing the four duties.

**Table 3:** Posture evaluation results of four tasks examined using 3DSSPP software.

		Mean ± SD/n (%)			
		Lifting mothers from bed	Breastfeeding training	Perineal suturing	Holding and handling newborns
Baby weight (kg)		–	2.991 ± 0.117	–	2.991 ± 0.117
Mother weight (kg)		70.109 ± 2.979	–	–	–
Sagittal plane low back analysis	Compression Forces at L_5_/S_1_(N)	2346.022 ± 417.246	1494.087 ± 268.639	1169.351 ± 343.090	485.989 ± 62.022
	Shear Forces at L_5_/S_1_ (N)	322.285 ± 35.155	244.802 ± 35.783	168.054 ± 45.005	160.758 ± 0.981
Joint Forces	C_7_/T_1_(N)	−59.382 ± 12.778	−51.585 ± 8.722	−39.685 ± 10.656	−84.142 ± 418.234
	Left Hand(N)	−68.340 ± 44.801	−14.967 ± 0.622	0	−14.967 ± 0.622
	Right Hand(N)	−50.703 ± 43.647	−14.967 ± 0.622	0	−14.967 ± 0.622
	Left Wrist(N)	−71.319 ± 49.454	−18.818 ± 0.911	−2.957 ± 0.795	−17.977 ± 0.622
	Right Wrist(N)	−58.947 ± 40.756	−18.818 ± 0.911	−2.961 ± 0.798	−17.581 ± 3.823
	Left Elbow(N)	−83.244 ± 49.804	−28.547 ± 2.391	−10.448 ± 2.805	−25.577 ± 0.622
	Right Elbow(N)	−68.222 ± 42.703	−28.503 ± 2.433	−10.447 ± 2.806	−25.533 ± 0.748
	Left Shoulder(N)	−103.415 ± 46.985	−46.171 ± 5.312	−1.632 ± 24.897	−39.3471 ± 0.622
	Right Shoulder(N)	−89.558 ± 40.235	−46.169 ± 5.315	−23.210 ± 8.927	−38.481 ± 8.276
	Left Hip(N)	−366.774 ± 279.468	−206.921 ± 57.338	−125.513 ± 54.234	−175.813 ± 2.853
	Right Hip(N)	−205.585 ± 279.205	−214.119 ± 57.802	−175.056 ± 47.893	−166.160 ± 2.783
	Left Knee(N)	−472.752 ± 279.742	−300.563 ± 46.116	−200.077 ± 52.862	−244.729 ± 2.851
	Right Knee(N)	−323.362 ± 268.751	−304.174 ± 52.384	−242.628 ± 66.398	−235.088 ± 2.774
	Left Ankle(N)	−510.861 ± 280.898	−331.564 ± 51.051	−223.084 ± 59.042	−268.094 ± 2.853
	Right Ankle(N)	−360.453 ± 267.046	−335.183 ± 57.309	−265.924 ± 72.299	−258.442 ± 2.782
Percent Capable	Wrist (%)	73.923 ± 20.593	100 ± 0	100 ± 0	100 ± 0
	Elbow (%)	80.527 ± 20.593	100 ± 0	100 ± 0	100 ± 0
	Shoulder (%)	76.989 ± 25.733	99.109 ± 0.314	100 ± 0	99.164 ± 0.373
	Torso (%)	76.527 ± 13.153	95 890 ± 1.601	98.142 ± 1.207	99.450 ± 0.500
	Hip (%)	36.549 ± 19.933	94.538 ± 2.217	96.637 ± 1.952	98.637 ± 0.483
	Knee (%)	49.89 ± 25.51	94.186 ± 9.404	100 ± 0	99.637 ± 0.483
	Ankle (%)	68.89 ± 30.573	96.175 ± 2.706	99.494 ± 0.750	100 ± 0
Balance	Acceptable (%)	76(83.5%)	91(100%)	91(100%)	91(100%)
	Critical (%)	15(16.5%)	–	–	–

The balance status of the midwives while lifting the mothers (from bed to sit or walk) was inappropriate and critical in 16.5% of the investigated cases due to the improper distribution of forces.

For the case of lifting the mothers from bed and helping them sit or walk, the results of this study (Table 3) showed that a lower percentage of the participants had no problem with using their seven major joints to perform this task. However, this was not the case for other tasks. Hips and knees were the limiting body parts and only less than 50% of the participating midwives had the required capability in these parts.

The shear force at the L_5_/S_1_ disc in all four duties was less than 500 N, which is in the safe range. For the case of lifting the mothers from bed and helping them sit or walk, the compression force at the L_5_/S_1_ disc was in the low-risk range for 97.8% of the study subjects, and in the medium-risk range for 2.2% of the study subjects.

The frequency distributions of risk levels for the compression forces at L_5_/S_1_ were evaluated in terms of age and work experience. The results showed that the highest frequency of risk was low for both variables. Routine events at work and home during the last seven days have more effect on MSDs, so the cumulative MSDs over the past 12 months were considered for the data analysis of the disorders.

Associations between forces acting on body parts and prevalence of MSDs in them.

The associations between individual factors and MSDs were analyzed using an independent *t*-test and chi-square statistical test with a significance level of p < 0.05. The results showed that age, height, body mass index, work experience, and marital status were significantly associated with pain in some anatomical body parts over the past 12 months. According to the fact that 54% of midwives had a BMI that was more than normal, statistical analysis showed that BMI has a significant relationship with musculoskeletal disorders in the Feet & Ankle area.

The associations between compression/shear forces at L_5_/S_1_ and MSDs in the upper and lower back regions were investigated using the independent *t*-test. The results showed that the Shear Forces at L_5_/S_1_ and the prevalence of MSDs in the upper and lower back regions are significantly associated while performing the task of holding and handling the newborns.

The associations between the forces at the joints and the prevalence of MSDs in each of the corresponding body parts while performing the four studied tasks were investigated. The results showed that for the perineal suturing posture, the MSDs in the left shoulder was significantly associated with the forces exerted on the corresponding joint. Moreover, for the task of holding and handling the newborns, the MSDs in the upper/lower back and the left pelvis were significantly associated with the forces exerted respectively on the L_5_/S_1_ and left pelvis joints (Table 4, Table 5).

**Table 4:** Prevalence of MSDs and the significance of the associations between MSDs and forces acting on different body parts in helping the mothers sit or walk and breastfeeding training.

Breastfeeding training					Helping the mothers sit or walk				
Body parts	GROUP	Mean of Force ± SD	T statistic	*P*-Value	Body parts	GROUP	Mean of Force ± SD	T statistic	*P*-Value
Compression Forces at L_5_/S_1_	Upper Back(59)	1468.983 ± 274.645	−1.214	0.228	Compression Forces at L_5_/S_1_	Upper Back(59)	2358.966 ± 416.557	0.4	0.96
	Upper Back(32)	1540.375 ± 254.91				Upper Back(32)	2322.156 ± 424.119		
Shear Forces at L_5_/S_1_	Upper Back(59)	242.661 ± 35.243	−0.773	0.441	Shear Forces at L_5_/S_1_	Upper Back(59)	324.678 ± 32.602	0.88	0.381
	Upper Back(32)	248.75 ± 36.995				Upper Back(32)	317.875 ± 39.601		
Compression Forces at L_5_/S_1_	Lower Back(52)	1498.769 ± 275.261	0.191	0.849	Compression Forces at L_5_/S_1_	Lower Back(52)	2377.480 ± 412.130	0.829	0.409
	Lower Back(39)	1487.846 ± 262.987				Lower Back(39)	2304.076 ± 425.686		
Shear Forces at L_5_/S_1_	Lower Back(52)	245.884 ± 36.121	0.332	0.741	Shear Forces at L_5_/S_1_	Lower Back(52)	325.769 ± 32.591	1.093	0.277
	Lower Back(39)	243.359 ± 35.745				Lower Back(39)	317.641 ± 38.245		
C_7_/T_1_	Neck (50)	−53.034 ± 5.589	−1.772	0.8	C_7_/T_1_	Neck (50)	−58.392 ± 17.185	0.815	0.417
	Neck (41)	−49.817 ± 8.657				Neck (41)	−60.591 ± 1.701		
Left Hand	Left Wrist &Hand (74)	−14.959 ± 0.65	0.241	0.81	Left Hand	Left Wrist &Hand (74)	−72.0 ± 43.958	−1.641	0.104
	Left Wrist &Hand (17)	−15 ± 0.5				Left Wrist &Hand (17)	−52.411 ± 46.285		
Right Hand	Right Wrist &Hand (71)	−14.929 ± 0.617	1.082	0.282	Right Hand	Right Wrist &Hand (71)	−49.957 ± 45.151	0.305	0.761
	Right Wrist &Hand(20)	−15.1 ± 0.64				Right Wrist &Hand(20)	−53.35 ± 38.78		
Left Wrist	Left Wrist &Hand (74)	−18.789 ± 0.932	0.631	0.529	Left Wrist	Left Wrist &Hand (74)	−74.258 ± 49.791	−1.186	0.239
	Left Wrist &Hand (17)	−18.944 ± 0.827				Left Wrist &Hand (17)	−58.524 ± 47.243		
Right Wrist	Right Wrist &Hand (71)	−18.805 ± 0.932	0.257	0.798	Right Wrist	Right Wrist &Hand (71)	−59.143 ± 41.691	−0.086	0.932
	Right Wrist &Hand(20)	−18.865 ± 0.853				Right Wrist &Hand(20)	−58.252 ± 38.26		
Left Elbow	Left Elbow (84)	−28.523 ± 2.395	0.326	0.746	Left Elbow	Left Elbow (84)	−82.123 ± 50.171	0.742	0.46
	Left Elbow(7)	−28.831 ± 2.51				Left Elbow(7)	−96.697 ± 46.447		
Right Elbow	Right Elbow (82)	−28.611 ± 2.413	−1.28	0.204	Right Elbow	Right Elbow (82)	−70.041 ± 43.913	−1.23	0.081
	Right Elbow (9)	−27.521 ± 2.535				Right Elbow (9)	−51.645 ± 25.44		
Left Shoulder	Left Shoulder (74)	−46.528 ± 5.323	−1.346	0.182	Left Shoulder	Left Shoulder (74)	−102.564 ± 47.038	0.359	0.721
	Left Shoulder (17)	−44.613 ± 5.129				Left Shoulder (17)	−107.119 ± 48.009		
Right Shoulder	Right Shoulder (67)	−46.372 ± 5.343	−0.606	0.546	Right Shoulder	Right Shoulder (67)	−90.203 ± 40.418	−0.254	0.8
	Right Shoulder (24)	−45.602 ± 5.307				Right Shoulder (24)	−87.756 ± 40.524		
Left Hip	Left Hip & Thigh (89)	−207.448 ± 57.874	−0.583	0.562	Left Hip	Left Hip & Thigh (89)	−367.916 ± 278.382	−0.259	0.796
	Left Hip & Thigh (2)	−183.47 ± 4.596				Left Hip & Thigh (2)	−315.95 ± 451.926		
Right Hip	Right Hip & Thigh (84)	−215.496 ± 59.257	−0.786	0.434	Right Hip	Right Hip & Thigh (84)	−208.501 ± 280.185	−0.343	0.732
	Right Hip & Thigh (7)	−197.587 ± 34.577				Right Hip & Thigh (7)	−170.604 ± 286.028		
Left Knee	Left Knee (77)	−302.466 ± 46.033	−0.922	0.359	Left Knee	Left Knee (77)	−486.337 ± 276.158	−1.088	0.28
	Left Knee (14)	−290.099 ± 46.857				Left Knee (14)	−398.03 ± 298.02		
Right Knee	Right Knee (76)	−303.433 ± 52.432	0.302	0.763	Right Knee	Right Knee (76)	−322.74 ± 273.58	−0.747	0.457
	Right Knee (15)	−307.928 ± 53.802				Right Knee (15)	−275.844 ± 245.885		
Left Ankle	Left Feet&Ankle (86)	−332.264 ± 50.869	−0.54	0.591	Left Ankle	Left Feet&Ankle (86)	−509.411 ± 283.641	0.203	0.84
	Left Feet&Ankle (5)	−319.534 ± 58.826				Left Feet&Ankle (5)	−535.796 ± 254.765		
Right Ankle	Right Feet & Ankle (84)	−336.089 ± 57.584	−0.521	0.604	Right Ankle	Right Feet & Ankle (84)	−363.949 ± 269.163	−0.431	0.668
	Right Feet & Ankle (7)	−324.3 ± 56.971				Right Feet & Ankle (7)	−318.504 ± 255.48		

*Correlation was significant at a level of 0.05.

**Table 5:** Prevalence of MSDs and the significance of the associations between MSDs and forces acting on different body parts in perineal suturing and holding and handling newborns.

Holding and handling newborns					Perineal suturing				
Body parts	GROUP	Mean of Force ± SD	T statistic	*P*-Value	Body parts	GROUP	Mean of Force ± SD	T statistic	*P*-Value
Compression Forces at L_5_/S_1_	Upper Back(59)	490.525 ± 63.858	0.947	0.346	Compression Forces at L_5_/S_1_	Upper Back(59)	1210.864 ± 370.477	1.580	0.089
	Upper Back(32)	477.625 ± 58.539				Upper Back(32)	1092.812 ± 274.987		
Shear Forces at L_5_/S_1_	Upper Back(59)	160.542 ± 1.039	−2.97	0.001*	Shear Forces at L_5_/S_1_	Upper Back(59)	173.966 ± 49.286	1.720	0.059
	Upper Back(32)	161.156 ± 0.723				Upper Back(32)	157.156 ± 33.857		
Compression Forces at L_5_/S_1_	Lower Back(52)	482.23 ± 61.235	−0.665	0.508	Compression Forces at L_5_/S_1_	Lower Back(52)	1172.480 ± 351.534	0.1	0.921
	Lower Back(39)	491.0 ± 63.506				Lower Back(39)	1165.179 ± 336.014		
Shear Forces at L_5_/S_1_	Lower Back(52)	160.519 ± 1.019	−2.871	0.005*	Shear Forces at L_5_/S_1_	Lower Back(52)	168.673 ± 46.492	0.15	0.881
	Lower Back(39)	161.076 ± 0.839				Lower Back(39)	167.230 ± 43.530		
C_7_/T_1_	Neck (50)	−120.094 ± 564.228	−0.905	0.368	C_7_/T_1_	Neck (50)	−39.651 ± 10.484	0.33	0.974
	Neck (41)	−40.30 ± 0				Neck (41)	−39.726 ± 10.993		
Left Hand	Left Wrist &Hand (74)	−14.959 ± 0.650	0.241	0.81	Left Hand	Left Wrist &Hand (74)	0	–	–
	Left Wrist &Hand (17)	−15.0 ± 0.50				Left Wrist &Hand (17)	0		
Right Hand	Right Wrist &Hand (71)	−14.929 ± 0.617	1.082	0.282	Right Hand	Right Wrist &Hand (71)	0	–	–
	Right Wrist &Hand(20)	−15.1 ± 0.640				Right Wrist &Hand(20)	0		
Left Wrist	Left Wrist &Hand (74)	−17.969 ± 0.650	0.241	0.810	Left Wrist	Left Wrist &Hand (74)	−2.979 ± 0.806	−0.548	0.585
	Left Wrist &Hand (17)	−18.01 ± 0.50				Left Wrist &Hand (17)	−2.861 ± 0.761		
Right Wrist	Right Wrist &Hand (71)	−17.432 ± 4.310	0.698	0.487	Right Wrist	Right Wrist &Hand (71)	−2.98 ± 0.801	−0.406	0.685
	Right Wrist &Hand(20)	−18.11 ± 0.640				Right Wrist &Hand(20)	−2.897 ± 0.803		
Left Elbow	Left Elbow (84)	−25.574 ± 0.629	0.145	0.885	Left Elbow	Left Elbow (84)	−10.472 ± 2.811	−0.3	0.765
	Left Elbow(7)	−25.61 ± 0.577				Left Elbow(7)	−10.141 ± 2.929		
Right Elbow	Right Elbow (82)	−25.549 ± 0.759	−0.611	0.543	Right Elbow	Right Elbow (82)	−10.571 ± 2.83	−1.276	0.178
	Right Elbow (9)	−25.387 ± 0.666				Right Elbow (9)	−9.318 ± 2.426		
Left Shoulder	Left Shoulder (74)	−39.312 ± 0.604	1.106	0.272	Left Shoulder	Left Shoulder (74)	−5.087 ± 25.163	−2.871	0.001*
	Left Shoulder (17)	−39.497 ± 0.696				Left Shoulder (17)	13.405 ± 17.392		
Right Shoulder	Right Shoulder (67)	−38.159 ± 9.634	0.618	0.538	Right Shoulder	Right Shoulder (67)	−23.096 ± 9.658	0.203	0.84
	Right Shoulder (24)	−39.380 ± 0.722				Right Shoulder (24)	−23.53 ± 6.635		
Left Hip	Left Hip & Thigh (89)	−175.877 ± 2.852	−1.431	0*	Left Hip	Left Hip & Thigh (89)	−125.894 ± 54.785	−0.445	0.657
	Left Hip & Thigh (2)	−172.975 ± 0.063				Left Hip & Thigh (2)	−108.56 ± 0		
Right Hip	Right Hip & Thigh (84)	−166.136 ± 2.787	0.285	0.777	Right Hip	Right Hip & Thigh (84)	−176.318 ± 48.020	−0.869	0.387
	Right Hip & Thigh (7)	−166.45 ± 2.943				Right Hip & Thigh (7)	−159.915 ± 47.098		
Left Knee	Left Knee (77)	−246.155 ± 2.160	−1.272	0.207	Left Knee	Left Knee (77)	−200.834 ± 54.098	−0.319	0.751
	Left Knee (14)	−244.628 ± 2.876				Left Knee (14)	−195.915 ± 47.057		
Right Knee	Right Knee (76)	−234.588 ± 2.707	0.494	0.622	Right Knee	Right Knee (76)	−246.773 ± 68.254	−1.347	0.124
	Right Knee (15)	−235.130 ± 2.791				Right Knee (15)	−221.627 ± 53.088		
Left Ankle	Left Feet&Ankle (86)	−266.53 ± 0	0.549	0.584	Left Ankle	Left Feet&Ankle (86)	−221.641 ± 58.738	0.966	0.337
	Left Feet&Ankle (5)	−268.111 ± 2.864				Left Feet&Ankle (5)	−247.888 ± 65.614		
Right Ankle	Right Feet & Ankle (84)	−258.422 ± 2.787	0.241	0.81	Right Ankle	Right Feet & Ankle (84)	−265.327 ± 72.398	0.271	0.787
	Right Feet & Ankle (7)	−258.687 ± 2.916				Right Feet & Ankle (7)	−273.082 ± 76.395		

*Correlation was significant at a level of 0.05.

## 4 Discussion

To the best of our knowledge, the present study presents the first estimation of the forces acting on the body parts of midwives while performing their duties and discusses the associations between these forces and MSDs among the midwives. According to the results of HTA and time analysis of the midwifery duties, midwives spend the largest portion of their working time in the following four activities: Holding and handling the newborns, perineal suturing, breastfeeding training, and helping the mothers sit or walk in the postpartum room.

According to the results, the percentage of the prevalence of MSDs among midwives is very high, and the highest prevalence corresponds to the back and neck regions. These results agree with the findings of previous studies conducted on midwives in England and Australia [11,12,16,25]. These results were expected since midwifery duties include manual and physical tasks, which require exerting force and involve awkward postures, such as bending, twisting, and extending the limbs, as well as static positions, which are difficult to avoid, especially during the birth process.

### 4.1 Postural analysis using 3DSSPP

In the present study, the forces on body parts during the four selected midwifery duties were estimated using 3D SSPP. The results showed that the highest compression and shear forces at L_5_/S_1_ were related to helping the mothers sit or walk. In the task of helping the mothers sit or walk, the compression force at L_5_/S_1_ was in the low- and medium-risk ranges, while the shear force was in the safe range. As observed, in the four studied tasks, the lowest force was exerted on the upper limbs, especially the hands and wrists, and the highest force was exerted on the lower limbs, especially the ankles. Moreover, only in the task of helping the mothers sit or walk, the balance status was inappropriate and critical due to the improper distribution of forces. In the task of helping the mothers sit or walk, hips and knees were the limiting body parts such that less than 50% of midwives had the required capability in the seven major joints parts to perform this task.

Okuyucu et al. used REBA posture assessment for midwives and showed that trunk postures were more at risk than other body parts. However, according to their results, the symptoms of MSDs in the knee and shoulder regions were also common [16].

Fiori et al. (2021) studied banknote printing workers using 3DSSPP. They estimated that the compression force at L_5_/S_1_ was between 1072 and 1863 N, and the shear force at this disc was between 263 and 310 N [26] Silvetti et al. (2020) conducted a study on curbside waste workers using 3DSSPP and showed that the workers balance was unacceptable in many tasks [27].

The 3DSSPP software was used in Refs. [[28], [29], [30]] to study biomechanical forces in manual load-carrying activities performed by women. The results of these references showed the risk of biomechanical overload and back injury. Ciaccia et al. (2020) used 3DSSPP to calculate the forces on the lower back and showed that the compression force on the back is significantly higher than the limit recommended by National Institute for Occupational Safety and Health (NIOSH) [29]. The participants in this study were women, and similar results were obtained for midwives in the tasks that involve carrying loads.

In other related studies on similar occupational groups, the weight of the patients is one of the main contributing factors to back injury in the task of manual carrying of loads [31,32]. When lifting the patient, the forces at L_5_/S_1_ can be affected by the patient's weight [33]. In agreement with the results of this study, another study reported that the forces at the L_4_/L_5_ joint are significantly high in the task of lifting the patient from the bed [34].

In the tasks of midwives, the weight of the patient, the height of the bed, and the bending/twisting of the body are among the ergonomic risk factors that are usually inevitable. However, many studies report the harmfulness of these risk factors for the spine, especially the lumbar region [35]. For example, the task of breastfeeding training exerts relatively high forces on the spine despite the low weight of the baby. This is probably due to the low bed, static posture, and bending and twisting of the body.

### 4.2 Associations between forces acting on body parts and prevalence of MSDs in them

In the present study, the associations between the forces exerted on body parts and the prevalence of MSDs among midwives were investigated. In agreement with previous studies on the association between lifting/moving loads in awkward postures and the prevalence of MSDs [9,36], in the task of handling newborns, the compression and shear forces at L_5_/S_1_ were significantly associated with MSDs in the past 12 months. Moreover, the forces exerted on the shoulder and pelvis were significantly associated with MSDs in the task of perineal suturing. The forces at the L_5_/S_1_ disc and pelvis were significantly associated with MSDs in these areas while handling newborns. The results of the present study were expected considering previous studies on the association between awkward postures and MSDs [8] and the effect of these postures on shear forces in the lumbar region.

Most midwives have to bend forward while performing their tasks. In this situation, the spine is twisted and tilted while the upper limbs are extended away from the trunk with respect to the sagittal axis. These results are also reported as risk factors for MSDs in another study on MSDs [3].

It is worth mentioning that the associations between the forces acting on different body parts and the prevalence of MSDs in these parts were not significant in some tasks. Among the midwives, the MSDs in some body parts such as the neck and back were reported to be high (i.e., 45.1% and 42.9%, respectively). Therefore, midwifery duties should be regarded as very important. According to these results, although the forces that are exerted on some body parts during midwifery tasks are not high and expose no risk, midwives may maintain a posture for a long time in many of their tasks. Grandjean recommended the maximum holding time (MHT) for a static posture at three levels based on the force required to maintain the posture. Accordingly, the recommended MHTs for a posture under large, medium, and small forces are 10 s, less than 1 min, and less than 4 min, respectively [37]. However, midwives have to maintain awkward postures much longer than 30 s during tasks such as perineal suturing and breastfeeding training.

Therefore, for future studies, it is suggested to evaluate the data on MHT of different postures in midwifery duties and compare the results with the existing ergonomic recommendations [38].

Ergonomic interventions, such as adjusting the workstation to the body dimensions and ergonomic training of employees, can somewhat reduce musculoskeletal discomfort by reducing biomechanical forces on the body. Such interventions, which are usually aimed at improving the postures of midwives during duties, are not sufficient, and the MHT of a static posture can also affect the occurrence of MSDs. However, no study has been conducted to address this issue among nurses and midwives.

## 5 Conclusion

Four midwifery duties including handling the newborns, perineal suturing, breastfeeding training, and lifting the mothers to sit or walk after delivery were analyzed using 3D SSPP. The results showed that the highest biomechanical forces were exerted on the spine at the L_5_/S_1_ disc during breastfeeding training. The biomechanical forces on the spine in each task could be affected by the weight of the mother, the height of the bed, static postures, and the bending and twisting of the body. Height, body mass index, and job tenure were among the risk factors that affected MSDs. However, it is worth mentioning that in some tasks, the associations between forces acting on different body parts and the prevalence of MSDs in these parts were not significant. According to these results, although the forces that were exerted on some body parts during midwifery tasks were not high and exposed no risk, midwives might maintain a posture for a long time in many of their tasks. Therefore, for future studies, it is suggested to evaluate the data on MHT of different postures in midwifery duties.

## 6 Limitations

The present study was conducted during the covid-referee pandemic, so the illness of the staff and the presence of fewer staff changed the shift work schedule. Accordingly, with the increase in the midwives’ workload, the rest period decreased, and it was possible that muscle recovery did not occur due to insufficient time. This may have affected the occurrence of MSDs. Examining all midwifery tasks in one study is not possible, and in this study we have only examined the most important tasks, other tasks can be studied in future studies. In this study, 91 midwives were examined, perhaps conducting a study on a larger number of midwives would affect the results of the study.

## Author contribution statement

Maryam Amirmahani: Performed the experiments; Wrote the paper.

Naser Hasheminejad: Contributed reagents, materials, analysis tools or data.

Somayeh Tahernejad: Conceived and designed the experiments; Analyzed and interpreted the data.

## Data availability statement

Data will be made available on request.

## Additional information

No additional information is available for this paper.

## Declaration of competing interest

The authors declare that they have no known competing financial interests or personal relationships that could have appeared to influence the work reported in this paper.
