# Supplementary material for: Biomechanical evaluation of midwifery tasks and its relationship with the prevalence of musculoskeletal disorders
Source: Heliyon. 2023 Aug 28;9(9):e19442. doi: 10.1016/j.heliyon.2023.e19442 (PMC10558586; doi:10.1016/j.heliyon.2023.e19442)
Supplement: Multimedia component 1 [file mmc1.docx]

Dear Editorial manager

I hope you are doing well!

In this study, the Persian version of the Nordic Musculoskeletal Disorders Questionnaire was used.

Choobineh et al evaluated the psychometric characteristics of the Persian version of NMQ[1, 2]

The questionnaire file is presented below.

Best regards,

Maryam Amirmahani

1. Choobineh, A., et al., *Musculoskeletal symptoms as related to ergonomic factors in Iranian hand-woven carpet industry and general guidelines for workstation design.* 2004. **10**(2): p. 157-168.

2. Kuorinka, I., et al., *Standardised Nordic questionnaires for the analysis of musculoskeletal symptoms.* Applied ergonomics, 1987. **18**(3): p. 233-237.

**با سلام و احترام**

**پیشاپیش از اینکه وقت خود را دراختیار اینجانب گذاشته و به سوالات زیر پاسخ می دهید بسیار سپاس گزارم.**

سن: .............. قد: .............. ورن: .............. سابقه کار: .............. وضعیت تاهل: مجرد متاهل

تحصیلات : کاردانی کارشناسی کارشناسی ارشد دکترا شغل دوم: بله خیر

وضعیت شیفت کاری: شیفت در گردش (نوبت کار) شیفت ثابت صبح (روزکار)

**وضعیت اسکلتی عضلانی**

1. **با توجه به شکل زیر نواحی از بدن خود را که در طول 7 روز گذشته در آنها احساس درد، ناراحتی یا بی حسی داشته اید مشخص کنید و شدت درد آن اندام را در قسمت مربوطه از جدول با یک عدد از 0 (بدون درد) تا 10 (شدیدترین حالت) مشخص کنید.**

| اندام | | درد شدید | جدول میزان درد | | | | | | | | | بدون درد |
| --- | --- | --- | --- | --- | --- | --- | --- | --- | --- | --- | --- | --- |
| 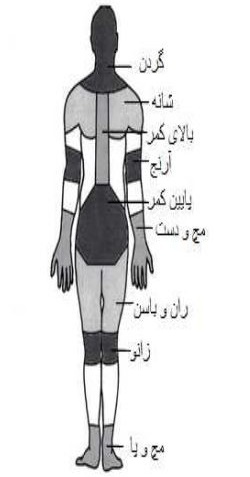 | گردن | 10 | 9 | 8 | 7 | 6 | 5 | 4 | 3 | 2 | 1 | 0 |
|  | شانه راست | 10 | 9 | 8 | 7 | 6 | 5 | 4 | 3 | 2 | 1 | 0 |
|  | شانه چپ | 10 | 9 | 8 | 7 | 6 | 5 | 4 | 3 | 2 | 1 | 0 |
|  | آرنج راست | 10 | 9 | 8 | 7 | 6 | 5 | 4 | 3 | 2 | 1 | 0 |
|  | آرنج چپ | 10 | 9 | 8 | 7 | 6 | 5 | 4 | 3 | 2 | 1 | 0 |
|  | مچ و دست راست | 10 | 9 | 8 | 7 | 6 | 5 | 4 | 3 | 2 | 1 | 0 |
|  | مچ و دست چپ | 10 | 9 | 8 | 7 | 6 | 5 | 4 | 3 | 2 | 1 | 0 |
|  | بالای کمر(پشت) | 10 | 9 | 8 | 7 | 6 | 5 | 4 | 3 | 2 | 1 | 0 |
|  | پایین کمر | 10 | 9 | 8 | 7 | 6 | 5 | 4 | 3 | 2 | 1 | 0 |
|  | باسن و ران راست | 10 | 9 | 8 | 7 | 6 | 5 | 4 | 3 | 2 | 1 | 0 |
|  | باسن و ران چپ | 10 | 9 | 8 | 7 | 6 | 5 | 4 | 3 | 2 | 1 | 0 |
|  | زانوی راست | 10 | 9 | 8 | 7 | 6 | 5 | 4 | 3 | 2 | 1 | 0 |
|  | زانوی چپ | 10 | 9 | 8 | 7 | 6 | 5 | 4 | 3 | 2 | 1 | 0 |
|  | پا و قوزک پای راست | 10 | 9 | 8 | 7 | 6 | 5 | 4 | 3 | 2 | 1 | 0 |
|  | پا و قوزک پای چپ | 10 | 9 | 8 | 7 | 6 | 5 | 4 | 3 | 2 | 1 | 0 |

1. **نواحی از بدن خود را که در طول 12 ماه گذشته در آنها احساس درد، ناراحتی یا بی حسی داشته اید مشخص کنید.**

گردن شانه راست شانه چپ آرنج راست آرنج چپ مچ و دست راست مچ و دست چپ بالای کمر(پشت) پایین کمر باسن و ران راست باسن و ران چپ زانوی راست زانوی چپ پا وقوزک پای راست پا وقوزک پای چپ

1. **نواحی از بدن که طی 12 ماه گذشته به دلیل مشکلات اسکلتی عضلانی از انجام فعالیت های روزمره نظیر فعالیت های شغلی، تفریحی و کار منزل بازمانده اید مشخص کنید.**

گردن شانه راست شانه چپ آرنج راست آرنج چپ مچ و دست راست مچ و دست چپ بالای کمر(پشت) پایین کمر باسن و ران راست باسن و ران چپ زانوی راست زانوی چپ پا وقوزک پای راست پا وقوزک پای چپ

**موفق باشید.**
